# Meta-analysis of internet-delivered interventions to increase physical activity levels

**DOI:** 10.1186/1479-5868-9-52

**Published:** 2012-04-30

**Authors:** Cally A Davies, John C Spence, Corneel Vandelanotte, Cristina M Caperchione, W Kerry Mummery

**Affiliations:** 1 Centre for Physical Activity Studies, Institute for Health and Social Science Research, CQ University Australia, Rockhampton, QLD, Australia; 2 Sedentary Living Lab, Faculty of Physical Education and Recreation, University of Alberta, Edmonton, AB, Canada; 3 Faculty of Health and Social Development, University of British Columbia, Kelowna, BC, Canada; 4 Faculty of Physical Education and Recreation, University of Alberta, Edmonton, AB, Canada

**Keywords:** Physical activity, Internet, Intervention, Meta-analysis

## Abstract

Many internet-delivered physical activity behaviour change programs have been developed and evaluated. However, further evidence is required to ascertain the overall effectiveness of such interventions. The objective of the present review was to evaluate the effectiveness of internet-delivered interventions to increase physical activity, whilst also examining the effect of intervention moderators. A systematic search strategy identified relevant studies published in the English-language from Pubmed, Proquest, Scopus, PsychINFO, CINHAL, and Sport Discuss (January 1990 – June 2011). Eligible studies were required to include an internet-delivered intervention, target an adult population, measure and target physical activity as an outcome variable, and include a comparison group that did not receive internet-delivered materials. Studies were coded independently by two investigators. Overall effect sizes were combined based on the fixed effect model. Homogeneity and subsequent exploratory moderator analysis was undertaken. A total of 34 articles were identified for inclusion. The overall mean effect of internet-delivered interventions on physical activity was *d* = 0.14 (*p* = 0.00). Fixed-effect analysis revealed significant heterogeneity across studies (*Q* = 73.75; *p* = 0.00). Moderating variables such as larger sample size, screening for baseline physical activity levels and the inclusion of educational components significantly increased intervention effectiveness. Results of the meta-analysis support the delivery of internet-delivered interventions in producing positive changes in physical activity, however effect sizes were small. The ability of internet-delivered interventions to produce meaningful change in long-term physical activity remains unclear.

## Introduction

Estimates from the World Health Organisation [1] suggest that approximately 60% of the world’s population are classified as inactive or insufficiently active to receive health benefits. With the increasing burden caused by physical inactivity and chronic disease, new ways for delivering behaviour change programs to large numbers of people at low cost are needed. In particular, the internet offers an innovative medium to produce health behaviour change in terms of reach, availability and opportunities for interactive approaches [2]. Statistics demonstrate more than a 300% increase in internet usage since 2000, with over 1.5 billion internet users worldwide, representing approximately 23.5% of the world’s population [3]. Most important, internet access provides an alternate means to health care promotion for individuals who cannot access standard care due to physical disability or living in remote areas [2]. Already a large number of individuals are utilising the internet to access health-related information [4], creating an opportunity to develop and deliver health-related behaviour change interventions via the internet. Furthermore, internet-delivered behaviour change interventions are becoming increasingly common for physical activity [2,5,6] particularly over the last 10 years [7].

Several reviews have examined the effectiveness of internet-delivered interventions to produce health related behaviour change among adults [2,6,7] and one has specifically examined the components and operationalisation of computer tailored programs across all ages [8]. Further, two meta-analysis were conducted on general health behaviours in both children and adults; one focusing on computer delivery [9] and another comparing web-based to non-web-based delivery [5]. The main findings indicate that: a) short-term behaviour change is more often reported than long-term behaviour change; [2,7-9] b) specific intervention elements such as website components (e.g., tailored content, theoretical design) and interactive features need to be further evaluated and explored for their role in both short-and long-term behaviour change and increasing website usage [2,6,7], and, c) internet-delivered physical activity interventions are more effective than true control groups [6]. This meta-analysis expands upon what is currently known through comprehensively synthesizing the effect of internet interventions on physical activity levels and variations in physical activity outcomes due to potential moderating variables. These findings will be useful to determine the current standing of internet interventions and to identify future directions for these types of interventions.

## Methods

### Inclusion criteria

To be included in the review, studies were required to provide an internet-delivered intervention with a focus on increasing physical activity. More specific, studies were included if they met the following criteria: a) participants were ≥ 18 years of age; b) the main form of intervention delivery was via the internet with either the use of a web page for the delivery and/or exchange of information, or in the form of email communication; c) physical activity and sample size measures were reported for both intervention and comparison groups; d) studies comprised of an experimental design, such as a randomized or quasi-experimental design; e) studies included a non-internet comparison group; and f) articles published in the English language. Studies that did not meet all inclusion criteria were deemed ineligible and were excluded. Additionally, studies that did not provide enough data to allow for the calculation of effect sizes were deemed ineligible.

### Search method

A comprehensive search strategy was undertaken to identify all possible studies for inclusion. The following electronic databases were searched: Pubmed, Proquest, Scopus, PsychINFO, CINHAL, and Sport Discuss. The search process was limited to articles published or provided ahead of publication access between January 1990 and June 2011. To locate potential studies the following search string was used: (“physical activity” OR exercise OR “physical fitness” OR walking) AND (internet OR “website delivered” OR “web based” OR,” world wide web”) AND (education OR behavio* OR intervention). All references including duplicates were then imported into EndNote (bibliographic software). Reference lists of all relevant review articles [2,5-13] were manually searched for potential studies not yet identified [14].

### Screening of articles

After the removal of duplicates, articles underwent two phases of screening to identify the final sample. Phase one involved scanning article abstracts for inclusion criteria to rule out literature that clearly did not meet the inclusion criteria. In phase two, full text versions of the remaining articles were obtained and further screened to identify the final set of articles for inclusion.

**Figure 1:**
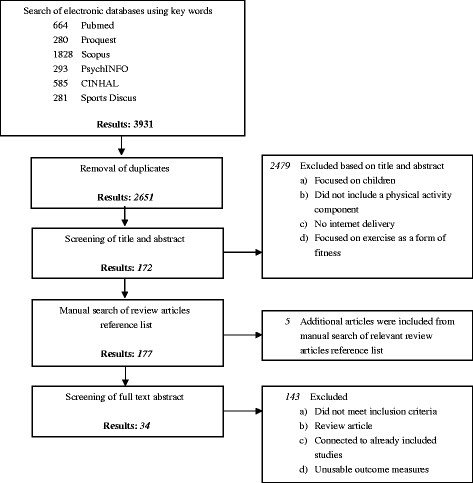
Selection for studies of internet delivered physical activity interventions.

The initial search strategy (excluding duplicates) identified 2651 potentially relevant articles. Following title/abstract screening and screening of relevant review articles, a reference list of 172 potentially relevant articles remained. After assessing the full text articles, the final set of articles for inclusion in this meta-analysis resulted in 34 primary articles (Figure 1) representing 34 unique interventions [15-48].

### Data extraction

Once finalised for inclusion, studies were collated and coded independently by two of the researchers (CD and CV), any discrepancies in coding were resolved through discussions [8,49]. The coding framework was pilot tested (i.e., both researchers independently coded two test articles) and refined prior to the first article being coded. Characteristics were coded under four general categories including: study design (e.g., sample size, physical activity mode targeted [i.e. leisure time or total], duration of intervention), participant characteristics (e.g., age, gender, population health status, and baseline physical activity levels), intervention features (e.g., number of intervention contacts, type of tailoring, presence of a theoretical underpinning, interactive features [e.g. goal setting, quizzes]) and intervention results (sample sizes, physical activity measures and any additional information to allow for the calculation of effect sizes). The list was developed based on previous reviews [2,5-8,10,50] and perusal of original research articles published on the topic. Articles were coded to provide descriptive information and to allow for subsequent moderator analysis. Tables 1 & 2 contain information of coded characteristics for the first three categories. The coding framework is not exhaustive of all intervention aspects and only characteristics reported in sufficient detail across studies are subsequently reported on.

**Table 1:** Study design and intervention characteristics

**Source**	**Country**	**Study Quality**	**Intention to Treat**	**Control Group**	**Baseline Sample**^**a**^	**Avg Age**^**a**^	**% Female**^**a**^	**Health Status**	**Additional Behaviours**	**Baseline Measure**	**Intro Session**	**Duration (weeks)**
Bosak and Yates, 2009 [15]	USA	Fair	Yes	SC	22	50.9	27	Metabolic Syndrome	Nil	Phone	Face	6
Carr et al.,2008 [16]	USA	Good	No	Control	67	45	81	Overweight	Nil	Face	Face	16
Cook et al.,2007 [17]	USA	Fair	No	Int	480	42.01	72	General	Nutrition	Internet	NR	12
Dunton and Robertson, 2008 [18]	USA	Good	No	Control	156	42.8	100	General	Nil	Internet	NR	12
Glasgow et al., 2010^b^[19]	USA	Good	Yes	SC	463	58.4	49.8	Diabetes	Self-M	Phone	Internet	16
Grim et al., 2011^b^[20]	USA	Fair	No	SC	233	21.2	72	General	Nil	NS	NR	10
Hager et al., 2002^b^[21]	USA	Fair	No	Control	525	42	56	General	Nil	Internet	NR	6
Haung et al., 2009 ^b^[22]	Taiwan	Good	No	Minimal	146	18	100	General	Nil	Face	NR	NR
Hurling et al., 2007 [23]	England	Fair	Yes	Control	77	40.4	67	General	Nil	Face	Face	9
Kim and Kang, 2006 ^b^[24]	South Korea	Good	No	SC	73	55.1	46.6	Diabetes	Nil	Face	Internet	12
Kosma et al., 2005 [25]	USA	Good	No	Control	151	38.7	72	Physical Disabilities	Nil	Internet	NR	4
Leibreich et al., 2009 [26]	Canada	Good	Yes	Minimal	49	54.1	59	Diabetes	Nil	Internet	NR	12
Lorig et al.,2006 [27]	USA	Good	No	SC	958	57.5	71.4	Chronic Disease	Self-M	Internet	NR	6
Lorig et al.,2008 [28]	USA	Good	No	SC	855	52.35	90.2	COPD	Self-M	Internet	NR	6
Lorig et al., 2010 [29]	USA	Good	Yes	SC	761	54.3	73	General	Self-M	Internet	NR	6
Mailey et al., 2011 [30]	USA	Good	No	SC	51	25	68.1	Mental Illness	Nil	Face	Face	10
Marshall et al., 2003 [31]	Australia	Good	Yes	Int	655	43	51	General	Nil	Phone	NR	8
McConnon et al., 2007 [32]	England	Fair	No	SC	221	45.8	77	Overweight	Weight loss	Face	Face	52
McKay et al., 2001 [33]	USA	Good	No	Minimal	78	52.3	53	Diabetes	Nil	Internet	Internet	8
Morgan et al., 2009 [34]	Australia	Fair	Yes	Control	65	35.9	0	Overweight	Weight loss	Face	Face	12
Morgan et al., 2011 [35]	Australia	Good	Yes	True	110	44.4	0	Overweight	Weight loss	Face	Face	14
Motl et al., 2011 [36]	USA	Good	No	Control	54	45.85	90	MultipleSclerosis	Nil	Mail	Internet	12
Napolitano et al., 2003 [37]	USA	Good	No	Control	65	42.8	86	General	Nil	Phone	NR	12
Nguyen et al., 2008 [38]	USA	Good	Yes	Int	50	69.5	44	COPD	Self-M	Face	Face	26
Ornes and Randsell, 2007^b^[39]	USA	Fair	Yes	Minimal	112	20.6	100	General	Nil	Face	Face	4
Parrott et al., 2008 [40]	USA	Good	Yes	Control	170	20.2	38	General	Nil	Face	NR	2
Plotnikoff et al., 2005 [41]	Canada	Good	No	Control	2121	44.9	73.5	General	Nutrition	Internet	NR	12
Skar et al., 2011 ^b^[42]	Scotland	Fair	Yes	True	1273	22.8	63.7	General	Nil	Internet	Internet	8
Smith et al.,2009 [43]	USA	Good	No	Control	41	43.5	80.5	Overweight	Nutrition	Face	Face	16
Spittaels et al.,2007 ^b^[44]	Belgium	Good	Yes	Control	434	42.4	66.1	General	Nil	Internet	NR	26
Steele et al., 2007 ^b^[45]	Australia	Good	Yes	Int	192	38.7	86	General	Nil	Face	NR	12
Wadsworth and Hallam, 2010 [46]	USA	Fair	No	Control	91	NS	100	General	Nil	Face	Face	26
Winnett et al.,2007 ^b^[47]	USA	Good	No	Control	1071	52.17	67	General	Nutrition	Face	NR	12
Zutz et al.,2007 [48]	Canada	Good	No	Control	15	58.5	20	Cardiac Rehab	Nutrition	Face	Face	12

Abbreviations: avg, average; COPD, chronic obstructive pulmonary disease; Self-M, self-management; Int, intervention; Intro, introductory; NR, not reported; SC, standard care.

^a^ If studies include more than two groups, information presented is inclusive of all groups included in the study; ^b^ Studies include more than two groups.

**Table 2:** Intervention features

**Source**	**Tailored**	**Theory**	**Interactive Features**	**Attrition (%)**		**Logins**	**Psyc Imp**
				**All**	**Int**		
Bosak and Yates, 2009 [15]	Limited	SCT	AC, Edu, ER, Fac, FB, GS, Q, SM, UC	14	17	NR	Yes
Carr et al., 2008 [16]	Limited	TTM	Edu, ER, Fac, FB, GS, Q, SM,	52	62	NR	NR
Cook et al., 2007 [17]	Nil	SCT, SOC	GS	13	15	NR	Yes
Dunton and Robertson, 2008 [18]	Full	TTM, HBM	Edu, ER, FB	15	16	NR	No
Glasgow et al., 2010^b^[19]	Nil	SCT, Self-M, SEM	Edu, ER, Fac, FB, GS, Q SM, UC	17	20	28	Yes
Grim et al., 2011^b^[20]	Nil	SCT,	Edu Q, UC	28	24	NR	Yes
Hager et al., 2002^b^[21]	Limited	TTM	FB	23	24	NR	Yes
Haung et al., 2009 ^b^[22]	Limited	TTM	AC, Edu, ER, FB, Q, SC, SM, UC	12	NR	NR	Yes
Hurling et al., 2007 [23]	Full	Other	AC, Edu, ER, FB, GS, SC,SM, UC,	NR	NR	26.1	Yes
Kim and Kang, 2006 ^b^[24]	Limited	TTM	AC, FB, GS, UC,	NR	NR	NR	No
Kosma et al., 2005 [25]	Limited	TTM	AC, Edu, FB, ER, UC,	50	54	NR	No
Leibreich et al., 2009 [26]	Limited	SCT	AC, Edu, ER, Fac, FB, SM, UC,	10	8	NR	Yes
Lorig et al., 2006 [27]	Limited	Self-M	AC, Edu, ER, Fac, FB, GS, Q, UC	19	22	26.5	No
Lorig et al., 2008 [28]	Full	Other	AC, Edu, ER, Fac, FB, GS, Q, SM, UC,	24	29	31.5	Yes
Lorig et al., 2010 [29]	Nil	Self-M	AC, Edu, ER, Fac, FB, GS, SC, SM, UC	15	20	NR	Yes
Mailey et al., 2011 [30]	NIL	SCT	FB, GS, SM, ER, UC, Edu	9	13	NR	Yes
Marshall et al., 2003 [31]	Limited	SOC	ER, FB, GS, Q	22	24	NR	NR
McConnon et al., 2007 [32]	Limited	Nil	ER, FB	31	51	15.8	NR
McKay et al., 2001 [33]	Full	Self-M, SEM	AC, Fac, FB, GS, SM	13	8	8.9	No
Morgan et al., 2009 [34]	Nil	SCT	AC, Fac, FB, GS, SM	17	18	120	NR
Morgan et al., 2011 [35]	Nil	SCT	Edu, FB, GS, SM	19	19	NR	Yes
Motl et al., 2011 [36]	Nil	SCT	AC, Edu, ER, Fac, FB, GS, SC, SM, UC,	11	15	8.6	Yes
Napolitano et al., 2003 [37]	Limited	SCT, SOC	Edu, ER, Q	12	30	NR	Yes
Nguyen et al., 2008 [38]	Full	SCT, Self-M, Other	Edu, ER, Fac, FB, GS, SC, SM	24	31	59	Yes
Ornes and Randsell, 2007^b^[39]	Nil	SCT	Edu, ER, FB, GS, SM,	7	NR	NR	NR
Parrott et al., 2008 [40]	Nil	TPB	AC	0	0	NA	Yes
Plotnikoff et al., 2005 [41]	Nil	SCT, TPB, TTM, PMT	Nil	18	NR	NA	Yes
Skar et al., 2011 ^b^[42]	Nil	TPB	FB	42	44	NR	Yes
Smith et al., 2009 [43]	Limited	TTM	Edu, ER, Fac, FB, GS, Q, SM	NR	NR	NR	NR
Spittaels et al., 2007 ^b^[44]	Full	TPB, SOC	AC, Edu, ER, FB, GS	34	40	NR	NR
Steele et al., 2007 ^b^[45]	Nil	SCT, Self-M	AC, Edu, ER, Fac, Q, SM, UC,	15	10	11.8	NR
Wadsworth and Hallam, 2010 [46]	Nil	SCT	Edu, ER, Fac, FB, GS, Q, SM, UC	22	24	NR	Yes
Winnett et al., 2007 ^b^[47]	Nil	SCT	Edu, FB, GS, SM, UC,	15	15	NR	No
Zutz et al., 2007 [48]	Nil	Nil	Edu, Fac, FB, Q, SC, UC	13	0	50	Yes

Abbreviations: Psyc Imp, psychological improvements; All, overall attrition for both groups; Int, attrition for internet intervention group only; NS, not reported; SCT, social cognitive theory, TTM, transtheoretical model; SOC, stages of change, HBM, health belief model; Self-M, self-management; SEM, social ecological model; TPB, theory of planned behaviour; PMT protection motivation theory; AC, asynchronous communication; Edu, education; ER, email reminders; Fac, facilitator; FB feedback; GS, goal setting; Q, quiz; SC, synchronous communication; SM, self-monitoring; UC, updated content,.

Note: Limited tailoring was defined as those interventions that mentioned the inclusion of some tailored materials, but did not deliver a comprehensively tailored intervention as the main component of the intervention. Presence of education material was defined as interventions that delivered structured educational material targeting physical activity knowledge. Psychological improvements are present where statistically significant improvements on any psychological measures is reported in the intervention group (e.g. self-efficacy, attitudes).

The majority of characteristics included in the first three categories, study design, participant characteristics and intervention features were also included in the moderator analysis. Potential moderating variables (see Table 3 for the list of moderator variables) were included if able to effectively extract data and code the variable. For example, the presence of a theoretical underpinning as a moderating variable of intervention effectiveness could not be examined, as only two studies did not specify a theoretical framework. Additionally, a number of continuous moderating variables were recoded into categorical variables to enable a more meaningful analysis to be undertaken. For example, although attrition was initially coded as a continuous variable it was re-coded as (1) above, or (2) below the average cross-study attrition rate of 23% for the internet intervention groups. Coding of articles resulted in an agreement rate of 92% between the researchers with any discrepancies being resolved.

**Table 3:** Summary statistics and effect sizes by moderator variable for change in physical activity as a result of internet-delivered interventions

**Variable**		**Q**_***b***_	***No.***	***d+***	**SE**	**95%CI**	**Q**_***W***_
*Study Design*							
Physical activity		0.07					73.68^a^
	Main outcome		25	0.14^a^	0.03	0.09/0.19	69.95^a^
	Secondary outcome		9	0.15^a^	0.04	0.07/0.23	3.73
Design		0.11					73.64^a^
	Randomised Trial		9	0.13^a^	0.04	0.05/0.21	25.36^a^
	Randomised Controlled Trial		25	0.16^a^	0.03	0.09/0.19	48.25^a^
Study Quality		0.47					73.28^a^
	Fair		10	0.13	0.05	0.02/0.20	29.18^a^
	Good		24	0.15^a^	0.02	0.10/0.20	44.10^a^
Sample Size		13.14^a^					13.14^a^
	<35 per group		15	0.40^a^	0.08	0.25/0.55	17.92
	≥35 per group		19	0.12^a^	0.02	0.07/0.16	42.70^a^
Physical Activity Mode		0.08					73.68^a^
	Leisure time		15	0.14^a^	0.03	0.08/0.19	20.32
	Overall		19	0.15^a^	0.04	0.08/0.22	53.36^a^
Additional Behaviours		0.05					73.70^a^
	No		21	0.15^a^	0.04	0.08/0.22	66.05^a^
	Yes		13	0.14^c^	0.03	0.08/0.19	7.65
Intervention Duration		2.01					66.72^a^
	0–6 weeks		8	0.11^a^	0.04	0.03/0.19	24.63^a^
	7–12 weeks		17	0.13^a^	0.03	0.08/0.19	35.45^a^
	13+ weeks		8	0.21^a^	0.06	0.09/0.33	6.65
Internet and/or Email		0.51					73.25^a^
	Internet and email		21	0.16^a^	0.04	0.09/0.23	34.12
	Only internet OR email		13	0.13^a^	0.03	0.08/0.18	39.13^a^
Comparison Group		10.50					63.25^a^
	Intervention group		4	0.03	0.06	−0.08/0.14	1.76
	Minimal intervention		4	0.43^a^	0.12	0.21/0.66	5.80
	Standard care		9	0.16^a^	0.04	0.09/0.23	23.23^a^
	Control group		17	0.14^a^	0.03	0.07/0.20	32.46
Intervention Attrition		4.59					39.82
	Below average (<23%)		16	0.16^a^	0.03	0.10/0.23	17.68
	Above average (>22%)		12	0.06	0.04	−0.01/0.13	22.14
*Participant Characteristics*							
Age		0.42					71.00^a^
	< 45 years		19	0.13^a^	0.03	0.07/0.18	46.38^a^
	> 44 years		14	0.15^a^	0.03	0.09/0.22	24.61
Gender		0.92					72.83^a^
	<60% female sample		12	0.10	0.05	0.01/0.19	39.57^a^
	>59% female sample		22	0.15^a^	0.02	0.10/0.20	33.26^a^
Health Status		4.13					69.62^a^
	General population		17	0.11^a^	0.03	0.06/0.17	44.06^a^
	Chronic diseased		12	0.19^a^	0.04	0.11/0.28	22.25
	Overweight		5	0.28^a^	0.11	0.07/0.48	3.31
Physical Activity Level		8.83^a^					64.92^a^
	Not screened for		25	0.12^a^	0.02	0.08/0.16	54.29^a^
	Sedentary		9	0.37^c^	0.08	0.21/0.52	10.63
*Intervention Features*							
Intervention Contacts		1.06					72.57^a^
	Less than 10		22	0.13^a^	0.03	0.07/0.18	63.03^a^
	10 or more		10	0.18^a^	0.04	0.10/0.25	9.54
Tailored		1.61					72.14^a^
	Comprehensive tailoring		6	0.13	0.06	0.02/0.24	1.92
	Limited tailoring		12	0.09	0.04	0.02/0.18	39.71^a^
	No tailoring		16	0.16^a^	0.03	0.11/0.22	30.51
SCT		6.85					66.91^a^
	Yes		16	0.20^a^	0.03	0.14/0.27	20.54
	No		18	0.09^a^	0.03	0.03/0.15	46.37^a^
TTM		0.80					72.95^a^
	Yes		9	0.11^a^	0.03	0.04/0.19	34.90^a^
	No		25	0.15^a^	0.03	0.10/0.21	38.05
Education Components		8.02^a^					65.73^a^
	Yes		24	0.20^a^	0.03	0.14/0.26	32.50
	No		10	0.08	0.03	0.01/0.14	33.23^a^
Goal Setting		1.05					72.70^a^
	Yes		19	0.16^a^	0.03	0.10/0.22	40.23^a^
	No		15	0.12^a^	0.03	0.06/0.12	32.47^a^
Self-Monitoring		3.85					69.91^a^
	Yes		18	0.20^a^	0.04	0.13/0.27	25.70
	No		16	0.11^a^	0.03	0.06/0.16	44.21^a^
Email Reminders		0.11					73.64^a^
	Yes		22	0.15^a^	0.03	0.09/0.21	34.27
	No		12	0.13^a^	0.03	0.07/0.19	39.37^a^
Updated Content		4.79					68.96^a^
	Yes		17	0.19^a^	0.03	0.13/0.26	34.68^a^
	No		17	0.10^a^	0.03	0.04/0.16	34.28^a^
Quizzes		0.10					73.66^a^
	Yes		12	0.15^a^	0.04	0.08/0.22	21.16
	No		22	0.14^a^	0.03	0.08/0.19	52.49^a^
Asynchronous Communication		0.58					
	Yes		15	0.16	0.04	0.09/0.23	32.37 ^a^
	No		19	0.13	0.03	0.08/0.18	40.79 ^a^

Abbreviations: Q_*b*_, heterogeneity between; No., number of studies; *d+*, weighted average effect size; SE, standard error; CI, confidence interval; Q_*w*_, heterogeneity within; SCT, social cognitive theory; TTM, transtheoretical model.

^a^ represents significance according to Bonferroni correction factor adjusted for each category (study design: *P* = < .005; participant characteristics: *P* = < .013 and intervention features *P* = < .005).

Study quality was assessed based on a previously developed methodological assessment tool, [51] which was modified to specifically address quality assessment for internet-delivered interventions [5]. This evaluation was based on five main criteria: a) study design; b) selection and specification of the study sample; c) specification of illness/conditions; d) reproducibility of the study; and e) outcomes specification and measurement. During the quality assessment process, studies could receive a score up to 18 points; the score obtained by each study was divided by 18 and multiplied by 100 to provide a “Study Quality Percentage”. Study Quality Percentages were then classified as good (66.7% or higher), fair (between 50 to 66.6%) and poor (less than 50%) [52].

### Data analysis

Effect sizes (*d*) were computed to represent the impact of the internet-delivered interventions on physical activity [53]. The effect size (*d*) is defined as the standardised mean difference and allows meaningful comparisons across measurement instruments. A positive effect size indicates a more favourable change in physical activity for the intervention condition. If studies reported statistics other than means and standard deviations (e.g., *Fp*), efforts were made to estimate *d* from the information provided [54]. For studies that used more than one follow up measure, effect sizes were calculated using data from the time point closest after intervention completion. This time-point was used in order to best determine the actual effects of the intervention. A separate effect size analysis was calculated for studies that reported six months or greater post-intervention follow up data; this was done to investigate the effectiveness of internet-delivered interventions in producing long-term behaviour change. To assess the possibility of publication bias the Egger test was used [55].

The fixed effects model was explored in relation to the summary effect size to estimate the mean distribution of effects. The heterogeneity statistic (*Q*) was calculated to determine whether studies shared a common effect size. *Q* represents the observed weighted sum of squares and *df* is the expected weighted sum of squares [53]. If heterogeneity was present among the effect sizes, further analysis was undertaken to examine study level moderating factors of physical activity outcomes [53]. The Bonferroni correction factor was applied to adjust the alpha value required for statistical significance within each of the three moderator categories (study design, participant characteristics, intervention features).

## Results

### Description of included studies

Articles were published from 2001 to 2011, with the modal year being 2007 (n = 8). Of the 34 studies, 21 were from the United States, 4 from Australia, 3 from Canada, 2 from England, and 1 each from Belgium, Taiwan, Scotland and South Korea. At baseline the 34 studies included a total sample size of 11,885 across all groups (including sample size from studies that included more than two groups) or 9,638 participants for the two comparison groups being examined in the current meta-analysis. Physical activity was the primary behaviour targeted in 25 (74%) of the studies.

The average duration of interventions was 12.64 weeks with a range of 2–52 weeks. Overall attrition (20%) was reported in 31 of the included studies with an attrition of 23% experienced in the intervention groups as reported in 28 studies. Although all studies used internet delivery, variation existed in the type of delivery employed. Specifically, 21 studies (62%) included a combination of internet and email, 9 (26%) used internet only and the remaining 4 (12%) used email only. The type of comparison group used also varied; with 26 studies (76%) using a true/standard care control group; while 4 studies used an alternate intervention (12%) and 4 studies used a minimal intervention comparison group (12%). In terms of study quality, none of the studies were rated as poor; 10 (29%) were rated fair, and 24 (71%) as good quality.

Nine (26%) studies used a sample population that were classed as inactive (not meeting the national physical activity guidelines according to their respective country recommendations). The other 25 articles (74%) did not screen for activity status. The majority of studies represented the general population (n = 17; 50%), with the remaining studies involving population with overweight (n = 5; 15%); Type 2 diabetes (n = 4; 11%); arthritis (n = 1; 3%); cardiac rehabilitation (n = 1; 3%); metabolic syndrome (n = 1; 3%); physical disabilities (n = 1; 3%); chronic disease (n = 1; 3%); multiple sclerosis (n = 1; 3%); a diagnosed mental illness (n = 1; 3%) and cardio obstructive pulmonary disorder (n = 1; 3%). The average age represented across studies was 43.06 years, 65% of the overall sample was female and, among the 18 articles that reported on ethnicity, 92% of the sample was Caucasian. The number of times participants logged in to the study website was reported for only 11 (32%) of the included studies. The average number of logins per person/per week was 3.08. Tables 2 & 3 outline study characteristics in more detail.

### Indication of results

**Figure 2:**
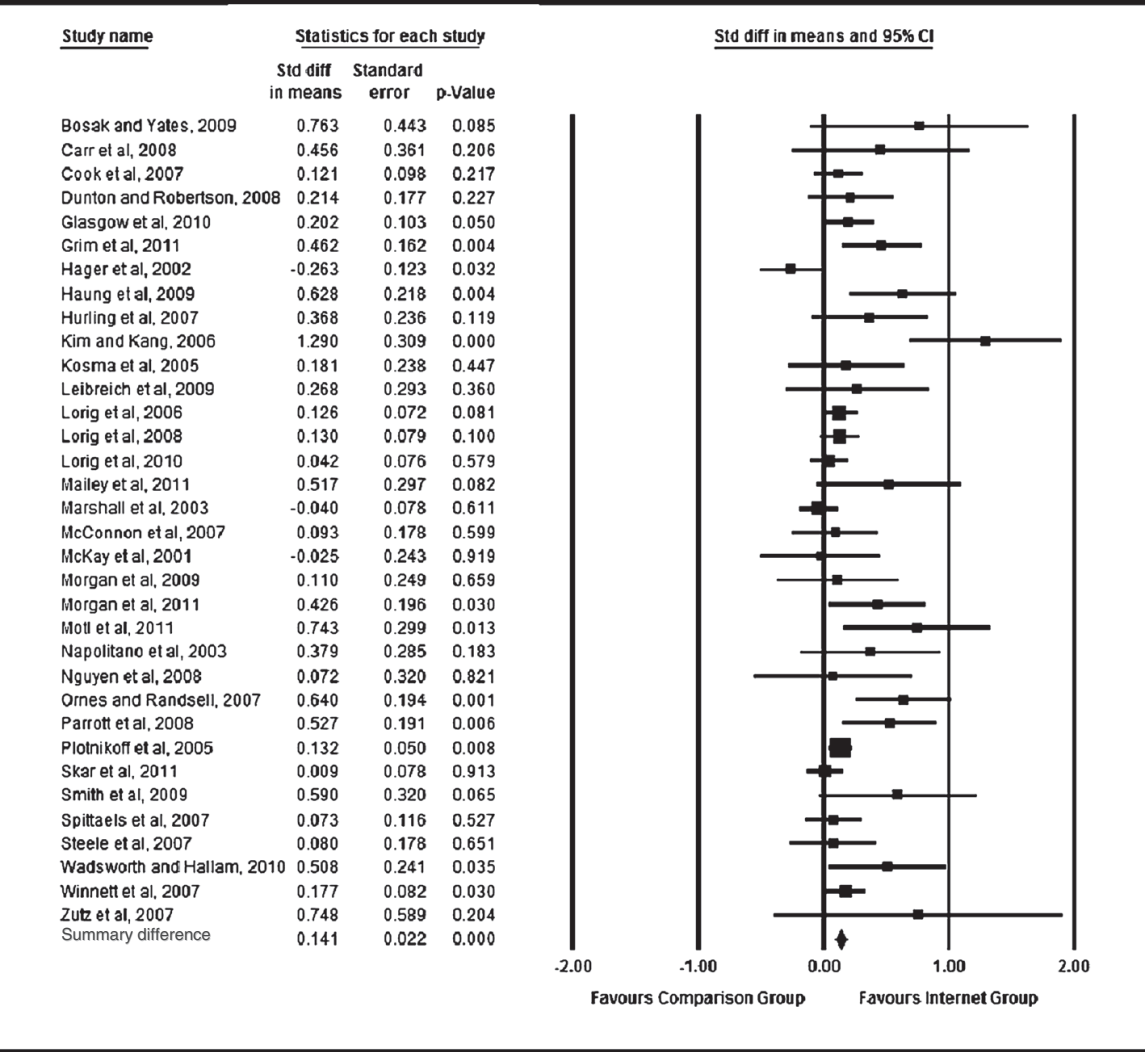
Forrest plot of effect sizes representing effect on physical activity behaviour.

The estimated overall mean effect of internet-delivered interventions on physical activity was *d* = 0.14 (*p* < 0.001; Figure 2), suggesting that internet-delivered interventions had a small but significantly greater impact on physical activity change than the comparison conditions. The results of the Egger test revealed that publication bias was present (*p* < 0.001). Thus, as recommended by Sterne and colleagues [56], no statistical methods were used to correct for publication bias, as corrections would be based on assumptions and therefore could produce potentially flawed results. Homogeneity tests from the fixed-effect analysis revealed significant heterogeneity across studies (*Q* = 73.75; *p* < 0.001). The overall mean effect for sustained physical activity at least 6 months post- intervention (n = 11) resulted in a small but significant effect size *d* = 0.11 (*p* < 0.01).

#### Study Design

Among the 10 study design variables (Table 3), post-intervention sample size (Q_b_ (1) = 13.1, *p* < 0.001) was the only significant moderator of internet-delivered interventions on physical activity. Specifically, studies that included less than 35 participants per group at the post-intervention follow up measure (*d* = 0.40) demonstrated larger effect sizes than the studies that had a post-intervention sample size of 35 or greater (*d* = 0.12).

#### Participant Characteristics

Of the four participant characteristic variables (Table 3), initial physical activity level (Q_b_ (1) = 8.83, *p* < 0.05) was found to be significant moderator of physical activity change. Studies that screened participants and included only participants classified as sedentary or insufficiently active produced a greater effect size (*d* = 0.37) than studies that did not screen participants for physical activity (*d* = 0.12).

#### Intervention Features

The inclusion of educational components was the only significant moderator (Q_b_ (1) = 8.02, *p* < 0. 005) of physical activity change among the 11 intervention features coded (Table 3). Specifically, interventions consisting of educational components producing a larger effect size (*d* = 0.20) than interventions that did not (*d* = 0.08).

## Discussion

The magnitude of the overall effect size indicates that internet-delivered programs have a small but positive effect on physical activity (*d* = 0.14). This finding is consistent with previous narrative reviews suggesting the ability of internet-delivered interventions to produce modest effects on physical activity [7,13]. The results also suggest these interventions produce variable effects on physical activity, as evident by the variance in effects across individual studies, which has also been highlighted by previous research [2,7,13]. Despite significant variability in intervention effects and the presence of possible moderator variables, the combined intervention outcomes resulted in improved short-term physical activity, with a smaller effect being found for longer-term behaviour change. This study presents the first meta-analytical review to extensively examine the impact of moderators on the effectiveness of internet-delivered interventions to increase physical activity.

The effect size is similar to recent findings from a meta-analysis investigating the effect of physical activity interventions for healthy adults across all modes of delivery [57]. The review did not specifically examine internet-delivered interventions but found the effect size across all delivery modes (*d* = 0.19) was similar to other mediated interventions (email and telephone) to increase physical activity levels (*d* = 0.15) [57]. However, the effect size was smaller than face-to-face interventions (*d* = 0.29) [57]. A previous review also attempted to conduct a meta-analysis on the effects of distance interventions to increase physical activity, but due to heterogeneity and poor study quality opted to do a systematic narrative review instead [50]. Since the cut-off date for the search strategy of that study [50], an additional 26 studies were published that were included in the current meta-analysis. Furthermore, due to the increase in publications and higher quality of these additional studies, it was possible to conduct a moderator analysis in the current study.

Internet-delivered interventions have the potential to produce small but significant increases in physical activity levels. Given the potential breadth of delivery, the public health impact of producing small changes in physical activity across a population has the potential for large positive changes at the population level [58]. This finding has even greater potential when considering the increase in effectiveness when specifically targeting a sedentary population (*d* = 0.37). Sedentary individuals are at higher risk of developing a number of chronic conditions and facing premature morbidity and mortality [1], hence it is encouraging to observe the effect sizes are larger for interventions targeting insufficiently active individuals. The potential implications for population level change remain tempered by questions as to whether these small effect sizes are clinically relevant. Additionally, the moderator analysis indicated that studies with a larger sample size have a smaller effect (*d* = 0.12) and it may be argued that these effect sizes are more representative of the actual effect of internet-delivered interventions on physical activity levels. Furthermore despite internet delivered interventions making claims about using the internet for reaching large populations at low cost, surprisingly few have evaluated their cost-effectiveness. We could not examine this factor in the current meta-analysis, and it is recommended that future studies evaluate the cost-effectiveness in terms of development, maintenance and breadth of delivery of internet-delivered interventions to allow comparisons to traditional modes of delivery.

Recently, meta-analyses examining the effectiveness of behavioural medicine interventions have been critiqued for including underpowered studies (less than 35 participants per group). It was argued that small studies are more likely to be published if they find positive results, increasing the likelihood of publication bias [55]. The outcomes of the present review confirm the presence of bias. Studies that do not find statistically significant results may not be being published either due to author’s not attempting to publish or journals not accepting the article for publication. Regardless, the findings should be interpreted within this limitation. There remains a need to include well-designed, randomised controlled trials that include adequate sample size at baseline to allow for attrition and to maintain sufficient power at the scheduled follow-up periods.

Increasing physical activity levels and maintaining the behaviour are important in terms of generating sustained health benefits [59]. Eleven studies followed participants for six months or more post-intervention. This resulted in a small effect size (*d* = 0.11) and further investigation is needed to determine the effectiveness of internet-delivered interventions to produce long-term change in physical activity levels. Future interventions should include long-term follow up measures for physical activity to identify overall effectiveness. Additionally, studies that targeted physical activity only or physical activity and additional behaviours produced similar effect sizes. This finding is supported by previous research [7] and provides justification for further investigation given the role of multiple behavioural risk factors in the development of non-communicable chronic disease.

Identifying factors that enhance intervention effectiveness can inform the development of future research to produce greater physical activity change. For instance, including structured educational materials that involved the exchange of information intended to influence physical activity was the only intervention feature found to moderate intervention effectiveness. Providing education has previously shown to be an effective behaviour change technique to increase physical activity among chronically ill adults [10]. Some of the intervention features examined, such as email reminders and updated content, are not necessarily included in an attempt to optimise intervention effectiveness but are incorporated as part of intervention design to enhance exposure to the program [60]. Previous research has demonstrated that intervention features such as the number of intervention contacts, tailored content, goal setting, self-monitoring and updated content enhance intervention effectiveness [7]. Although these intervention features were not found to be significant moderators on physical activity change in the current analysis, future research should attempt to isolate the impact of specific intervention features on physical activity change through implementing high quality study designs (such as randomised controlled trials, having adequate sample sizes, using validated instruments to measure study outcomes and appropriate reporting of results) that will allow such investigation.

Based on 11 of the included studies, the average number of logins was 3.08 per-person-per-week which exceeds the traditional one-contact–per-week that is common among face-to-face interventions. Due to lack of data, it was not possible to analyse the decline in website logins over time, however it is an issue often identified throughout the literature [7,60]. Several of the studies that did track decay of logins over time have reported that the majority of intervention logins occurred within the first few weeks of the study duration with a very steep decline shortly thereafter [31,61,62], hence it is an important issue. Enhancing participant engagement is directly related to increased exposure to the intervention and research has identified a clear dose–response relationship between the intensity of the intervention and resulting behaviour change [61,63]. It is therefore apparent that maintaining website engagement is an important factor in relation to the potential effectiveness [31,33,61,64,65]. Due to limited reporting of login and other website engagement data, the impact of intervention features on program engagement could not be evaluated. Results from a recent systematic review suggest that intervention features, such as provisions for peer or counsellor support, email and/or phone contact with visitors and regular website updates, were related to increased exposure in internet-delivered healthy lifestyle interventions [60]. However, the authors noted significant issues in the consistency of reporting engagement measures and recommended that future interventions apply consistent engagement measures across studies [60]. Consistent with the current study, Brouwer and colleagues [60] identified that the most common measure of website engagement was the average number of logins to the intervention website, therefore future web-based interventions should also consistently report on it. Nevertheless, additional measures of website engagement are required, such as decline in website logins over time and time exposed to specific intervention elements.

### Limitations

The results presented should be interpreted within the limitations of the current meta-analysis. Due to the low number of articles we were unable to combine variables and conduct a meta-regression when examining the impact of moderators. This is important as features that were significant moderators also displayed significant heterogeneity, which indicated the presence of other factors still influencing the effect size. In this respect moderator analysis should be interpreted with caution. A number of features were also unable to be examined as part of the moderator analysis due to insufficient reporting among primary articles. Another limitation is that the majority of studies mostly incorporated the use of self-report measures for physical activity, included largely Caucasian and well education samples. Although the majority were based on valid and reliable measures, the better measure is still to use objective measures. In terms of internet delivery this can prove challenging if programs are being widely disseminated. Finally, the effect size cannot be translated to represent a more meaningful and clinically relevant change in physical activity level (for example minutes of moderate or vigorous physical activity) as studies vary widely in the form of measured used for assessing behaviour.

## Conclusion

Overall, the findings demonstrate internet-delivered interventions are effective in producing small but significant increases in physical activity. Although the effects are small, producing such changes in behaviour across a large population can have powerful implications at a population level. To fully harness the potential of internet-delivered interventions to produce population-wide effects it is important that interventions target insufficiently active individuals as well as more diverse populations. Programs are continuing to evolve with advances in technology, but it is imperative that rigorous high quality research continues to explore the effectiveness of internet delivered interventions. Future research in the area should focus on the various aspects of internet-delivered interventions that increase the engagement and retention of the target audience so as to better understand the elements that will enhance effectiveness of this type of intervention.

## Competing interests

The authors declare that they have no competing interests to disclose.

## Authors’ contributions

CD led the search strategy and was assisted by CV in screening and coding articles. CD, JS and CV were responsible for the analysis and interpretation of the data. All authors participated in the study design, drafting of the manuscript and critical revisions. All authors read and approved the final manuscript.
